# A strategic blueprint for strengthening respiratory syncytial virus prevention among under-five children in low- and middle-income countries: Bangladesh as a model for new immunisation approaches

**DOI:** 10.7189/jogh.16.03013

**Published:** 2026-06-05

**Authors:** Saju Bhuiya, Fahmida Chowdhury, Md Ariful Islam, Tanzir Ahmed Shuvo, Mohammad Abdul Aleem, Ahamed Khairul Basher, Homayra Rahman Shoshi, Ashrak Shad Pyash, Mofakhar Hussain, Tahmina Shirin, Mahmudur Rahman, Nusrat Homaira, Md Zakiul Hassan

**Affiliations:** 1 International Centre for Diarrhoeal Disease Research, Bangladesh, Dhaka, Bangladesh; 2 School of Population Health, University of New South Wales, Sydney, New South Wales, Australia; 3 Institute of Health Policy, Management and Evaluation, University of Toronto, Ontario, Canada; 4 Institute of Epidemiology, Disease Control and Research, Dhaka, Bangladesh; 5 The Eastern Mediterranean Public Health Network, Dhaka, Bangladesh; 6 Discipline of Paediatrics and Child Health, School of Clinical Medicine, University of New South Wales Medicine and Health, University of New South Wales, Sydney, New South Wales, Australia; 7 Respiratory Department, Sydney Children’s Hospital, University of New South Wales, Randwick, Sydney, Australia; 8 James P. Grant School of Public Health, BRAC University, Dhaka, Bangladesh; 9 Nuffield Department of Clinical Medicine, University of Oxford, Oxford, UK; 10 International Severe Acute Respiratory and emerging Infection Consortium, University of Oxford, Oxford, UK

## Abstract

Respiratory syncytial virus (RSV) is the leading cause of respiratory morbidity and mortality in children under five years of age, especially in low- and middle-income countries (LMICs). In 2023, two RSV immunisation options, including a maternal RSV vaccine and a monoclonal antibody (mAb), became approved in several high-income countries. The World Health Organization has recently prequalified the maternal vaccine and the Gavi has committed funding for its introduction, raising the prospect of RSV vaccine availability in LMICs in the near future. This analysis thus underscores the need for country-specific data on the burden of disease and seasonality, as well as the acceptability of new immunisation options and evaluations of their cost-effectiveness. Achieving successful implementation will require health system strengthening, targeted strategies to address unwillingness to vaccinate, securing sustainable financing for immunisation, and ensuring sustained policy commitment. As in many other LMICs, the RSV infection continues to pose a substantial public health concern in Bangladesh. However, evidence gaps persist regarding RSV epidemiology, healthcare system readiness, and public perceptions and willingness regarding new immunisation options. We use Bangladesh as a model to identify gaps, review available evidence, and outline strategies for integrating maternal vaccine and infant mAb into the country’s maternal and child health programmes. Lessons drawn from this case study may be directly applicable to other LMICs, offering a pathway to equitable adoption of these life-saving interventions and reducing the substantial global burden of RSV.

Respiratory syncytial virus (RSV) remains a leading cause of acute lower respiratory infections (ALRIs) among children under five years of age, contributing to 33 million infections, 3.6 million hospitalisations, and 26 300 deaths annually, with the highest burden in the first six months of life [1–3]. Moreover, global data indicate that more than 97% of RSV-related deaths occur in low- and middle-income countries (LMICs) [1–3]. Prior to 2023, palivizumab, a high-cost humanised monoclonal antibody, available mostly in high-income countries, was the only immunoprophylaxis against severe RSV disease for high-risk children [4]. However, two new effective immunisation options, including maternal active immunisation with the RSVpreF vaccine (Abrysvo), a protein subunit vaccine, and passive immunisation with monoclonal antibodies (mAb) (nirsevimab), have been approved in multiple high-income settings. However, use of these two new immunisation approaches remains limited in LMICs, where the burden of RSV is the highest.

In March 2024, the World Health Organization (WHO) prequalified the maternal RSV vaccine, enabling United Nations procurement for LMICs, while a coalition of 44 organisations urged Gavi to accelerate access to RSV immunisation for children under five years of age [1]. Gavi has since prioritised RSV prevention and, in July 2025, its Programme and Policy Committee recommended establishing a funding window for maternal RSV immunisation, pending Gavi Alliance Board approval [1]. Nevertheless, the successful implementation of these immunisations options in LMICs will require well-coordinated, context-specific strategies aligned with national health systems and epidemiological priorities.

While RSV immunisation in high-income countries is often promoted on the basis of its potential to reduce seasonal hospitalisations and associated healthcare costs, the context in LMICs such as Bangladesh is more complex. Although the country’s RSV burden remains inadequately characterised, available evidence suggests it accounts for ~20% of hospitalised respiratory infections among children under five years of age, with an in-hospital mortality rate of about 2% [2–9]. Bangladesh’s constrained health system, in which bed occupancy rates exceeded 164–175% in 2025, further limits its capacity to manage seasonal surges [10]. Families also have to spend up to ~USD 100 per illness episode (~24% of monthly household income), with many forced to borrow money for care [11]. These clinical, system, and economic pressures highlight the potential value of maternal RSV vaccination and infant monoclonal antibodies to reduce infant morbidity and mortality, ease health system strain, and reduce financial hardship.

This viewpoint, therefore, aims to identify evidence gaps and provide recommendations for implementing RSV immunisation strategies to strengthen RSV control among children under five years of age in Bangladesh. The opinions expressed here stem from our own direct involvement in the surveillance of several influenza and other respiratory viruses and long-term engagement with vaccinology and vaccine effectiveness studies. We identified the literature informing this viewpoint through repeated, unstructured purposive searches of PubMed and Google Scholar using broad terms related to RSV, immunisation, and Bangladesh. We prioritised publications from the last 25 years and sought to include diverse sources, including randomised trials, observational data, qualitative studies, modelling analyses, and expert consensus statements, to capture both established evidence and emerging signals (Table S1 and S2 in the **Online Supplementary Document**). Then, using the WHO’s Evidence-to-Recommendation (EtR) Framework, we assessed the potentiality of introducing maternal RSV vaccination and infant mAb interventions in Bangladesh which is also adaptable in other LMICs.

## ETR DOMAIN #1 – PRIORITY OF THE PROBLEM

### Epidemiology, seasonality, genotypes, and economic burden

RSV is one of the leading causes of ALRI in children under five years of age in Bangladesh [4,7]. A recent hospital-based study reported a 30% RSV positivity among patients with acute respiratory infection (ARI), with rates of 36% among patients hospitalised with severe acute respiratory infection (SARI) and 16% among those taken into ambulatory care for influenza-like illness [12]. The burden is higher among children aged <6 months compared with other age groups [12]. Another hospital-based study reported a 20% positivity among patients meeting the extended SARI case definition, with the highest positivity again observed among children aged <6 months [8] A study investigating in-hospital deaths among patients with SARI found that 12–13% of those who died were RSV infected [6]. Research also suggests that early winter and winter months are the RSV circulation season [4,5]. Moreover, RSV onset starts in the southern districts in August and spread to the central and northern regions during winter [12]. Data indicated a predominance of RSV-A in the pre-COVID-19 pandemic period [13] and the RSV-B group during and after the pandemic (specifically the clade B.D.5.2.1.1) [9].

The economic burden of RSV in Bangladesh is also poorly understood. A study conducted over a decade ago estimated the median direct hospitalisation costs at USD 62 and indirect costs at USD 19 per episode, corresponding to annual direct costs of approximately USD 10 million [11]. These figures should be interpreted as historical estimates, rather than decision-grade inputs, given inflation, changes in household income, rising hospital costs, and evolving care-seeking pathways. The lack of robust national surveillance and cost-effectiveness analyses thus prevents a clear assessment of the public health impact of RSV in Bangladesh.

### Health system readiness and implementation gaps:

Several system-level challenges in Bangladesh hinder the effective implementation and impact of new RSV immunisation approaches. While healthcare facilities, excluding pharmacies, demonstrate moderate to high general service readiness [14], the country receives the lowest scores of all LMICs for infection prevention and control due to suboptimal cold chain storage in peripheral areas resulting from disrupted electricity supplies and a lack of temperature monitoring during vaccine transportation [14–16]. There are also no data on parental awareness or provider readiness for maternal RSV vaccination; evidence from other LMICs shows that uptake may be limited by low health literacy, provider misconceptions, and financial or sociocultural barriers [17]. Furthermore, the acceptability of RSV maternal vaccines and infant mAb in Bangladesh remains unexplored, underscoring the prerequisite for targeted research and community engagement to ensure equitable and effective implementation of RSV prevention strategies. Diagnostic testing, meanwhile, is available only in a few private hospitals in Dhaka and is absent from public tertiary facilities, hindering early case detection and limiting physician awareness of the RSV disease burden, thus weakening the foundation for vaccine introduction in Bangladesh [18]. Finally, vaccine implementation research is lacking. Lessons from the introduction of pneumococcal conjugate vaccine in Bangladesh highlight the importance of ongoing surveillance, political commitment, supply chain readiness, and community engagement [19]. Equivalent preparation for RSV is yet to be undertaken.

## ETR DOMAIN #2 – BENEFITS AND HARMS OF THE INTERVENTION

### Current landscape for immunisation of severe RSV disease among children under five years of age

Until recently, prophylaxis was limited to a high-cost monoclonal antibody palivizumab, administered monthly over five doses and available only for high-risk children under five years of age [20]. In 2023, a maternal vaccine and a mAb were approved in several high-income countries for this population after demonstrating strong effectiveness against morbidity and mortality [21]. Additionally, clesrovimab, another long-acting monoclonal antibody, was approved by the US Food and Drug Administration in 2025, while the WHO prequalified the maternal vaccine in 2024 and the Gavi has proposed a funding window for its introduction in LMICs (**Table 1**) [9–11].

**Table 1:** RSV medical countermeasures: vaccines, monoclonal antibodies immunoprophylaxis for children under five years of age

Category	Name	Target Population	Dose schedule	Administration	Approval status by national regulatory agency	WHO prequalification	WHO/SAGE policy status	Gavi financing status	Efficacy/cost	Availability in Bangladesh
Vaccines	Abrysvo (Pfizer)	Older adults and pregnant women (to protect children under five years of age) [22]	Single 0.5 mL dose	Intramuscular [22]	US FDA & EMA approved (2023) [22]	March 2025 [1]	Issued recommendations in September 2024 [1]	Proposed a funding window post-prequalification [1]	Vaccine efficacy of 84.4% (95% CI = 59.1–95.2) among persons aged ≥60 years [22]; approximate cost USD 295 per dose	Unavailable
mAb	Beyfortus (Nirsevimab)	All children under five years of age entering their first RSV season [21]	Single dose	Intramuscular [21]	US FDA (2023), EMA (2022), and MHRA approved (2022), EU (2022), UK (2022), Canada (2023) [21]	Not yet	Recommended	Under consideration	83.2% efficacy in preventing RSV hospitalisations in children under five years of age [21]; USD 519.75 per dose for 50mg and 100mg doses	Unavailable
	Synagis (Palivizumab)	High-risk children under five years of age (preterm, congenital heart disease, chronic lung disease) [20]	15 mg dose every 28 d during RSV season (4–5 doses)	Intramuscular [20]	US FDA approved (1998) [20]	Not yet	Long-standing use in high-risk groups	Not applicable	55% reduction in RSV-related hospitalisations; [20]; ~ USD 1910 for a supply of 0.5 mL dose [23]	Unavailable
	Clersovimab-CFOR (Enflonsia)	Neonates and children under five years of age born during or entering their first RSV season (aged <12 mo) [24]	Single dose	Intramuscular	Under FDA review; PDUFA target date 10 June 2025 [24]	Not applicable	No WHO policy yet	Not applicable	~ 84% reduction in RSV-associated hospitalisations and ~ 90% reduction in RSV-related lower respiratory infection hospitalisations [24]	Unavailable

AAP – American Academy of Pediatrics, CI – confidence interval, EMA – European Medicines Agency, FDA – Food and Drug Administration, MALRTI – moderate to severe acute lower respiratory infection, MHRA – Medicines and Healthcare products Regulatory Agency, PDUFA – Prescription Drug User Fee Act, RSV – respiratory syncytial virus, SAGE – Strategic Advisory Group of Experts on Immunization, TBD – to be determined, WHO – World Health Organization

## ETR DOMAIN #3 – VALUES AND PREFERENCES

Stakeholder values and preferences are likely to play a pivotal role in the uptake and effectiveness of RSV immunisation strategies in Bangladesh. When new interventions are endorsed by government authorities and integrated into routine contacts, acceptance among caregivers has historically been favourable, suggesting an enabling environment for RSV immunisation [25].

**Figure 1:**
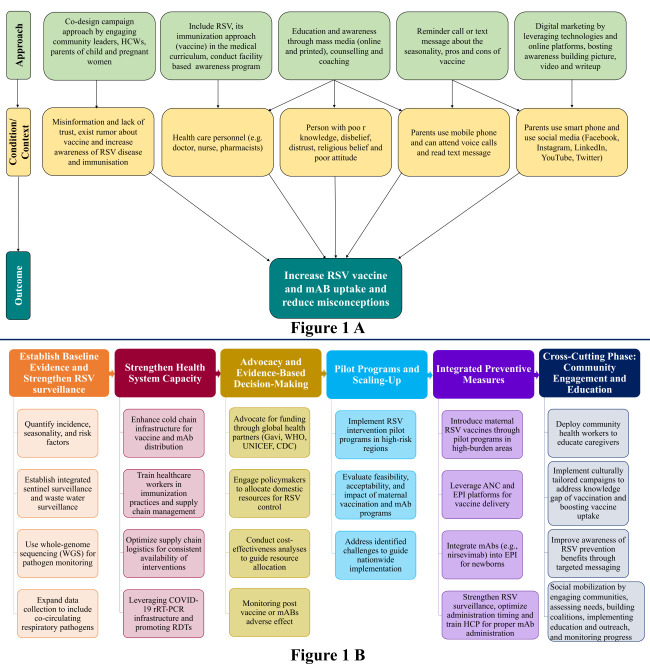
The multifaceted approach to increasing vaccine uptake (**Panel A**) and implementing RSV immunisation approaches (**Panel B**) in LMICs such as Bangladesh. ANC – antenatal care, EPI – Expanded Programme on Immunization, HCP – healthcare providers, RSV – respiratory syncytial virus, WHO – World Health Organization.

Addressing vaccine-related misconceptions and improving public awareness is vital to successful RSV immunisation efforts. A recent study found that only 3% of parents in LMICs were aware of RSV, underscoring the need for targeted awareness-raising interventions [3], such as strategic media campaigns which have driven high vaccine uptake in countries like Argentina and Australia [26,27]. Aside from media, advocacy groups and local champions play a key role in dispelling myths and building trust [26,27]. Nationwide communication about RSV immunisation, its benefits, and potential side effects is essential for increasing acceptance. In Bangladesh, low literacy levels may hinder understanding of scientific messages, making culturally tailored communication crucial. These community healthcare workers, with deep community ties, are well-positioned to promote maternal vaccination and mAb programmes [28]. Our proposed pathway outlines tailored interventions based on local conditions and population needs, offering a framework to reduce vaccine-related misconceptions and support equitable access to RSV prevention [29] (**Figure 1**, Panel A).

Despite some limitation, Bangladesh is well-positioned to integrate RSV immunisations into its existing healthcare system through the Expanded Programme for Immunization (EPI). Coordinated by the Directorate General of Health Services (DGHS) of Bangladesh, the EPI has consistently achieved high coverage for vaccines such as measles and polio by leveraging a robust network of community health workers and primary healthcare facilities [25]. To guide implementation, we propose a multifaceted framework informed by global vaccination strategies and tailored for Bangladesh (**Figure 1**, Panel B) [30,31]. Nevertheless, experience from previous EPI vaccination programme expansions indicates that support for adoption may weaken in the absence of adequate staffing, training, and reliable supply chains.

## ETR DOMAIN #4 – ACCEPTABILITY AND STAKEHOLDERS

The acceptability of RSV preventive interventions in Bangladesh is likely to be favourable among key stakeholders, including caregivers, healthcare providers, and policymakers, although direct empirical evidence is limited. No studies have specifically assessed perceptions of RSV immunisation among pregnant women or young children in Bangladesh. Evidence from other settings suggests generally positive attitudes; for example, maternity professionals in the UK have expressed support for routine RSV vaccination [32]. Although population-level evidence on values and preferences is scarce, maternal and childhood vaccination programmes in Bangladesh have historically achieved high coverage, supported by the EPI and routine maternal tetanus vaccination services. However, without clear communication, caregivers may underestimate the benefits of RSV prevention. Acceptability is also shaped by social and household decision-making processes, where husbands, senior family members, and community leaders often influence maternal and child health decisions [33]. Engagement with these stakeholders and transparent communication regarding safety and benefits will be important for sustaining public trust. Healthcare providers and programme implementers are likely to view RSV preventive interventions favourably if they reduce seasonal hospital admissions and pressure on paediatric services. Successful implementation will require institutional support, adequate resources, and clear delivery frameworks.

## ETR DOMAIN #5 – RESOURCE USE AND ECONOMIC CONSIDERATIONS

The economic burden of RSV in Bangladesh is also poorly understood. A study conducted over a decade ago estimated annual direct costs of approximately USD 10 million [12]. Updated micro-costing studies across public and private sectors, incorporating household financial risk and forgone care, are urgently needed to inform financing discussions and cost-effectiveness modelling. Moreover, to date, no cost-effectiveness modelling on the RSV vaccines or mAb has been conducted in Bangladesh, while global modelling indicates maternal vaccination and infant mAb are cost-effective in LMICs when considering the number of disability-adjusted life years averted and reduced hospitalisations [34].

The WHO prequalification and Gavi prioritisation create opportunities for procurement and support LMICs [1]. Engaging key national stakeholders – the National Immunization Technical Advisory Group, the Inter-Agency Coordinating Committee, the Ministry of Health and Family Welfare, the DGHS, parliamentarians – is vital to securing consensus and formal adoption into the EPI. Public–private partnerships can boost delivery and advocacy, while alignment with global priorities (*e.g.* WHO, Gavi, UNICEF) could attract economic and technical support to RSV vaccination in Bangladesh [35]. National ownership and innovative financing are key to building resilient, self-sustaining immunisation systems that can respond to future health threats and reduce disease burden.

## ETR DOMAIN #6 – EQUITY

Equity considerations are central to RSV immunisation decisions in Bangladesh, as the burden of severe disease and its financial consequences are unevenly distributed. Children under five years of age from low-income households, rural or hard-to-reach areas, and families with limited care-seeking capacity face higher risks of delayed treatment and severe outcomes [8,12]. Geographic disparities in access to oxygen therapy, paediatric beds, and referral systems further increase vulnerability. Integrating maternal vaccination or mAb administration into the national EPI and routine antenatal care (ANC) platforms could improve equity by expanding access across socioeconomic groups. Public financing, targeted outreach to underserved areas, strengthened cold-chain systems, and community-based risk communication will be essential for ensuring equitable coverage and avoid widening disparities.

## ETR DOMAIN #7 – FEASIBILITY

Bangladesh has substantial experience in delivering both maternal and infant immunisation through established platforms, offering a solid foundation for RSV intervention. ANC services, outreach vaccination sessions, and national cold-chain systems provide entry points that could be leveraged for rapid deployment. Previous introductions of new vaccines demonstrate the programme’s capacity to adapt guidelines, conduct training cascades, and achieve high coverage within relatively short timeframes. However, several feasibility challenges require attention. Pharmacovigilance systems must be prepared to monitor safety in pregnant women and young under five years of age, populations that typically receive heightened scrutiny. Regulatory approval pathways, procurement planning, and sustainable financing arrangements could also influence the pace of introduction. Human resources also warrant consideration; frontline workers are already managing multiple priorities, and adding counselling responsibilities for a new intervention may strain consultation time. Clear eligibility criteria, simple delivery algorithms, and integration into existing reporting structures will help minimise burden.

## RECOMMENDATION: ROADMAP FOR INTEGRATION OF RSV CONTROL MEASURES FOR PREVENTING SEVERE RSV OUTCOME AMONG CHILDREN UNDER FIVE YEARS OF AGE

### Establish baseline evidence and strengthen RSV surveillance

Building a robust evidence base is the foundation for RSV control and evaluating its health impact in Bangladesh. According to the WHO, no single system can fully capture the complex dynamics of respiratory virus surveillance. Instead, a ‘mosaic’ of integrated surveillance systems and complementary studies is essential to comprehensively assess the risk, transmission, severity, and impact of respiratory viruses with epidemic potential, such as RSV [34]. As such, Bangladesh should establish year-round sentinel surveillance integrated with existing influenza and COVID-19 monitoring systems with the collaboration of the DGHS, the Institute of Epidemiology, Disease Control and Research (IEDCR), and the icddr,b [36]. As an early warning system, integrated wastewater surveillance, initially developed for COVID-19, could also be an alternative option for identifying community-level RSV infection [37]. In addition, systematic data on cost, vaccine efficacy, and long-term outcomes must be generated to inform cost-effectiveness modelling [38]. Given current data limitations, simplified static models could be used for initial cost-effectiveness analyses, since passive immunisation is unlikely to affect RSV transmission [39]. Such analyses would help estimate cost-effectiveness and project RSV-associated hospitalisations, deaths averted, and as well as the associated economic benefits, thereby providing useful evidence to guide national policy decisions. Lastly, surveillance of RSV sequences across various platforms, geographic regions, and temporal contexts will be crucial for establishing baseline data and understand the true RSV burden in Bangladesh [40].

### Near-term (0–12 months): strengthen health system capacity, enhance health system readiness, and ensure efficient supply chain management

It will be prudent to leverage the EPI infrastructure of Bangladesh for cold chain maintenance of timely delivery of maternal RSV vaccine or mAB. Investing in solar-powered cold chain equipment and mobile temperature monitoring systems to ensure reliable vaccine storage and transportation, particularly in peripheral areas, while improving infection prevention and control training for healthcare workers, will also help strengthen immunisation delivery mechanism [41]. Institutions such as the IEDCR and icddr,b are well-positioned to lead the development of RSV-specific training modules to build workforce readiness for future RSV vaccine and mAb introduction. Enhancing RSV testing facilities by leveraging existing COVID-19 rRT-PCR infrastructure and expanding point-of-care can enhance RSV detection, optimise clinical management, reduce inappropriate antibiotic use, and inform targeted immunisation to mitigate RSV infection, transmission and morbidity in children under-5 in LMICs like Bangladesh [1,33]. Finally, data suggest that non-pharmaceutical interventions was associated with a substantial reduction in RSV incidence as well as other respiratory virus infection, which is very much convenient option to reduce transmission in the non-vaccinated area (Table S3 in the **Online Supplementary Document**) [42].

### Medium-term (1–3 years): piloting and scaling up intervention programme

Pilot programmes and strategic scale-up are essential for introducing RSV immunisation in LMICs like Bangladesh. Understanding willingness to pay and participate across socioeconomic groups is critical for tailoring effective strategies [31]. Pilots should target high-risk regions to assess the feasibility, acceptability, and impact of maternal vaccination and infant mAb programmes, while addressing implementation challenges [43]. Leveraging country-specific capacities and platforms like HBIS, and with support from WHO, US CDC, and the Gates Foundation, an RSV surveillance system integrated with WHO's Global Influenza Surveillance and Response System (GISRS) can be piloted to generate data on disease burden, seasonality, and high-risk populations. These efforts provide a scalable, cost-effective framework for guiding vaccination and prophylaxis strategies [44]. Scaling up based on pilot findings can help LMICs overcome barriers such as limited access and awareness, while seasonally targeted approaches enhance efficiency. Ultimately, well-designed pilots will inform evidence-based policies and ensure equitable access to RSV interventions, reducing disease burden in Bangladesh and similar settings.

### Long term (will be continued) integrating maternal vaccines and mAb into existing immunisation system:

Maternal RSV vaccines have shown efficacy in reducing severe RSV cases in children under five years of age, without any safety concerns identified [45]. This evidence is supported by clinical trials and studies conducted in several countries, many of which have already integrated maternal RSV vaccination into their national immunisation programmes [46]. Moreover, the WHO’s EtR framework analysis suggests that the desirable effects of maternal RSV vaccination and infant RSV mAb are substantial, while the undesirable effects are minimal to moderate, supporting their introduction over no vaccination (Table S4 in the **Online Supplementary Document**) [47].

Routine ANC visits offer a practical and scalable opportunity for vaccine delivery, especially in resource-limited settings. In Bangladesh, maternal RSV vaccination could be effectively integrated into the existing ANC framework, which includes at least four routine visits. Leveraging current infrastructure for maternal tetanus immunisation could facilitate efficient rollout. When available in Bangladesh, maternal RSV vaccination could be introduced through a step-by-step approach (Figure S1 in the **Online Supplementary Document**).

The mAb offers promising protection from RSV-related lower respiratory tract infections for children under five years of age, particularly those born preterm who may not benefit from a maternal RSV vaccine [21,48]. The integration of mAb into EPI programmes of Bangladesh could enhance newborn protection. Importantly, no adverse effects have been identified for the co-administration of maternal vaccine to the mother and the mAb to the infant after birth, or for other routine childhood vaccines [49]. To effectively monitor post-vaccination adverse events, LMICs like Bangladesh can leverage existing platforms such as the Adverse Events Following Immunization system managed by the DGHS to systematically collect and analyse adverse event reports [50].

## CONTEXTUALISING BANGLADESH AS A STRATEGIC PROTOTYPE FOR RSV IMMUNISATION APPROACH INTRODUCTION

Bangladesh is conceptualised in this analysis not as a universal template for all LMICs, but as a strategic prototype for immunisation transition settings. The country combines strong routine immunisation performance under the EPI, established respiratory pathogen surveillance platforms, functional ANC services, a hybrid delivery architecture managed by public institutions and non-governmental organisations, and rapid urbanisation with a concentrated population of children under five years of age, making it an analytically relevant case for examining operational pathways for RSV prevention in comparable LMIC contexts. Several elements of this approach are potentially transferable to structurally similar settings, including the leveraging of existing EPI delivery platforms, the integration of maternal RSV vaccination into routine ANC, the adoption of an influenza surveillance platform for RSV monitoring, and the implementation of phased introduction strategies beginning in high-burden urban areas. However, transferability is contingent upon comparable health system capacity, financing trajectories, and policy prioritisation. Context-specific features such as high urban density, mature district-level microplanning, strong integration between the Government and non-governmental organisations, and an evolving Gavi co-financing pathway reflect Bangladesh’s distinct political economy of health and may not be generalisable to fragile or lower-coverage systems. Accordingly, Bangladesh is framed as a bounded policy laboratory for high-density, immunisation-mature LMICs, while other contexts may require phased system strengthening prior to adopting similar RSV introduction strategies.

## CONCLUSIONS

The introduction of RSV maternal vaccines and infant mAb offers a pivotal opportunity to reduce RSV-related morbidity and mortality in Bangladesh. The country is well-positioned to integrate RSV prevention into existing maternal and child health platforms such as EPI and ANC, ensuring seamless and sustainable delivery. However, success will depend on addressing critical gaps. With strategic alignment across sectors, Bangladesh not only safeguards its most vulnerable populations, but also sets a powerful example for other LMICs. By embracing comprehensive year-round surveillance, targeted implementation research, and robust economic evaluations, the country can unlock the full potential of these life-saving immunisation approaches. A unified, multi-sectoral strategy grounded in strong data systems, community engagement, and public trust will be instrumental in driving success. Collaborative efforts among government agencies, academia, public health leaders, and private-sector partners will be key to generate evidence, ensure sustainable financing and develop effective policy framework. By establishing a context-specific, evidence-driven pathway, Bangladesh can ensure equitable access, financial sustainability, and operational excellence in RSV prevention. Addressing these priorities offers Bangladesh the chance not only to reduce RSV morbidity and mortality, but also to contribute evidence that can guide regional and global strategies.

## Additional material


Online Supplementary Document
