# Supplementary material for: A strategic blueprint for strengthening respiratory syncytial virus prevention among under-five children in low- and middle-income countries: Bangladesh as a model for new immunisation approaches
Source: J Glob Health. 2026 Jun 5;16:03013. doi: 10.7189/jogh.16.03013 (PMC13237809; doi:10.7189/jogh.16.03013)
Supplement: Online Supplementary Document [file jogh-16-03013-s001.pdf]

Supplement to: Bhuiya S, Chowdhury F, Islam MA, Shuvo TA, Aleem MA, Basher AK, Shoshi HR, Pyash AS, Hussain M, Shirin T, Rahman M, Homaira N, Hassan MZ. A strategic blueprint for strengthening respiratory syncytial virus prevention among under-five children in low- and middle-income countries: Bangladesh as a model for new immunisation approaches. J Glob Health. 2026;16:03013.

Table S1. Reviewed Evidence Sources by Type, Country Income Group, and Purpose

S.N.	Reference No(s).	Document Type	Country Income Group	Primary Purpose of Review
1	1–4, 22, 28, 29, 35, 51, 52	Systematic reviews / Multicountry epidemiology / Surveillance analyses	Global / Mixed income	Global RSV burden, epidemiology, seasonality patterns, surveillance gaps
2	5, 7, 24, 71	National policy guideline / Clinical directive	HIC (UK, Australia, USA)	RSV prophylaxis policies, maternal vaccination guidance, infant immunisation recommendations
3	6	Clinical review	HIC (USA)	Therapeutic options and clinical management of RSV
4	8–11	Vaccine efficacy / Equity commentary / Clinical trials	Global / Mixed income	Maternal vaccine and monoclonal antibody effectiveness, equity implications
5	12, 13, 48, 62, 67	Global policy frameworks / Gavi / WHO guidance	Global	RSV introduction strategy, investment case, Evidence-to-Recommendation frameworks
6	14–21, 25–27, 30, 70	Country-specific epidemiology and burden studies	LMIC (Bangladesh)	RSV burden, hospitalisation, mortality, surveillance data
7	31–33, 37, 43, 69, 72	Immunisation system performance / EPI guidelines	LMIC (Bangladesh, Africa, Asia) + Global	Cold chain readiness, EPI coverage, AEFI systems, immunisation platform strength
8	34, 73–75	Maternal immunisation / Vaccine hesitancy studies	Global / LMIC	Determinants of vaccine uptake, hesitancy mitigation strategies
9	36	Regional burden assessment	HIC (Europe)	Health system burden and service impact of RSV

S.N.	Reference No(s).	Document Type	Country Income Group	Primary Purpose of Review
10	38–42, 55, 68	Regulatory approval / Pricing / Cost-effectiveness / Safety	HIC (USA, Europe, Canada)	Product approval, pricing, procurement, vaccine safety, economic evaluation
11	44–47, 50, 53, 54, 60, 61	Surveillance innovation / Public health resilience / Influenza analogues	Global / LMIC (Bangladesh, Asia)	Lessons from COVID-19, surveillance strengthening, influenza platform adaptation
12	45	National policy analysis	LMIC (Bangladesh)	Influenza policy lessons applicable to RSV introduction
13	49	WHO surveillance methodology	Global	Estimating RSV disease burden using WHO surveillance platforms
14	56	Economic modelling methodology	HIC (Europe)	Static vs dynamic modelling approaches in cost-effectiveness analysis
15	57, 58	Cold chain logistics and management	HIC (UK) & LMIC (India)	Vaccine storage, cold-chain system management
16	63, 64	Public–private partnership analyses	LMIC (Africa, Asia)	Private sector financing and immunisation delivery models
17	65	Costing study	LMIC (Kenya)	Health system cost of RSV immunisation delivery
18	66	RSV prophylaxis strategy analysis	LMIC (Africa)	Public health considerations for RSV prevention in African settings

Reviewed Documents:

1 Karron RA, Black RE. Determining the burden of respiratory syncytial virus disease: the known and the unknown. Lancet. 2017;390(10098):917–8.

2 Du Y, Yan R, Wu X, Zhang X, Chen C, Jiang D, et al. Global burden and trends of respiratory syncytial virus infection across different age groups from 1990 to 2019: A systematic analysis of the Global Burden of Disease 2019 Study. Int J Infect Dis. 2023;135:70–6.

3 Li Y, Wang X, Blau DM, Caballero MT, Feikin DR, Gill CJ, et al. Global, regional, and national disease burden estimates of acute lower respiratory infections due to respiratory syncytial virus in children younger than 5 years in 2019: a systematic analysis. Lancet. 2022;399(10340):2047–64.

4 Respiratory syncytial virus infection among children younger than 2 years admitted to a paediatric intensive care unit with extended severe acute respiratory infection in ten Gavi-eligible countries: the RSV GOLD-ICU Network study. Lancet Glob Health. 2024;12(10):e1611–e9.

5 Department of Health and Social Care UK. Palivizumab passive immunisation against Respiratory Syncytial Virus (RSV) in at risk pre-term infants. United Kingdom: NHS, UK; 2021.

6 Turner TL, Kopp BT, Paul G, Landgrave LC, Hayes D Jr., Thompson R. Respiratory syncytial virus: current and emerging treatment options. Clinicoecon Outcomes Res. 2014;6:217–25.

7 Australian Government. Respiratory syncytial virus (RSV). Australiya: Australian Government; 2025.

8 Pérez Marc G, Vizzotti C, Fell DB, Di Nunzio L, Olszevicki S, Mankiewicz SW, et al. Real-world effectiveness of RSVpreF vaccination during pregnancy against RSV-associated lower respiratory tract disease leading to hospitalisation in infants during the 2024 RSV season in Argentina (BERNI study). Lancet Infect Dis. 2025.

9 Drysdale SB, Cathie K, Flamein F, Knuf M, Collins AM, Hill HC, et al. Nirsevimab for Prevention of Hospitalizations Due to RSV in Infants. N Engl J Med. 2023;389(26):2425–35.

10 Plock N, Sachs JR, Zang X, Lommerse J, Vora KA, Lee AW, et al. Efficacy of monoclonal antibodies and maternal vaccination for prophylaxis of respiratory syncytial virus disease. Commun Med (Lond). 2025;5(1):119.

11 Barsosio HC, Bont LJ, Groome MJ, Karron RA, Kragten-Tabatabaie L, Madhi SA, et al. How Gavi support for RSV immunisation will advance health equity. Lancet. 2025.

12 Gavi tVA. Gavi welcomes first-ever prequalification of a maternal RSV vaccine. 2025.

13 Committee TGAPaP. Report to the Board: Respiratory Syncytial Virus (RSV) Investment Case. 2025.

14 Homaira N, Luby SP, Hossain K, Islam K, Ahmed M, Rahman M, et al. Respiratory Viruses Associated Hospitalization among Children Aged <5 Years in Bangladesh: 2010–2014. PLoS One. 2016;11(2):e0147982.

15 Reller ME, Mehta K, McCollum ED, Ahmed S, Anderson J, Roy AD, et al. Viral Acute Lower Respiratory Tract Infections (ALRI) in Rural Bangladeshi Children Prior to the COVID-19 Pandemic. Influenza Other Respir Viruses. 2024;18(12):e70062.

16 Saha S, Saha S, Kanon N, Hooda Y, Islam MS, Islam S, et al. Health-care burden related to respiratory syncytial virus in a resource-constrained setting. Lancet Glob Health. 2025;13(6):e1072–e81.

17 Begum MN, Karim Y, Jubair M, Khair S, Tony S, Patwary M, et al. High Burden of Respiratory Syncytial Virus Among Bangladeshi Hospitalized Children Under Five. 2024.

18 Directorate General of Health Services. Real Time Health Information Dashboard. Government of Bangladesh; 2024.

19 Bhuiyan MU, Luby SP, Alamgir NI, Homaira N, Sturm-Ramirez K, Gurley ES, et al. Costs of hospitalization with respiratory syncytial virus illness among children aged <5 years and the financial impact on households in Bangladesh, 2010. J Glob Health. 2017;7(1):010412.

20 Stockman LJ, Brooks WA, Streatfield PK, Rahman M, Goswami D, Nahar K, et al. Challenges to evaluating respiratory syncytial virus mortality in Bangladesh, 2004–2008. PLoS One. 2013;8(1):e53857.

21 Hassan MZ, Islam MA, Haider S, Shirin T, Chowdhury F. Respiratory Syncytial Virus-Associated Deaths among Children under Five before and during the COVID-19 Pandemic in Bangladesh. Viruses. 2024;16(1).

22 Kim L, Rha B, Abramson JS, Anderson LJ, Byington CL, Chen GL, et al. Identifying Gaps in Respiratory Syncytial Virus Disease Epidemiology in the United States Prior to the Introduction of Vaccines. Clin Infect Dis. 2017;65(6):1020–5.

23 Buonsenso D. Bivalent Prefusion F Vaccine in Pregnancy to Prevent RSV Illness in Infants. N Engl J Med. 2023;389(11):1053.

24 Government of United Kingdom. RSV vaccination of pregnant women for infant protection: information for healthcare practitioners. 2025.

25 Chowdhury F, Shahid A, Ghosh PK, Rahman M, Hassan MZ, Akhtar Z, et al. Viral etiology of pneumonia among severely malnourished under-five children in an urban hospital, Bangladesh. PLoS One. 2020;15(2):e0228329.

26. Nasreen S, Luby SP, Brooks WA, Homaira N, Al Mamun A, Bhuiyan MU, et al. Population-based incidence of severe acute respiratory virus infections among children aged <5 years in rural Bangladesh, June-October 2010. PLoS One. 2014;9(2):e89978.

27. Haque F, Husain MM, Ameen KM, Rahima R, Hossain MJ, Alamgir AS, et al. Bronchiolitis outbreak caused by respiratory syncytial virus in southwest Bangladesh, 2010. Int J Infect Dis. 2012;16(12):e866-71.

28. Haynes AK, Manangan AP, Iwane MK, Sturm-Ramirez K, Homaira N, Brooks WA, et al. Respiratory syncytial virus circulation in seven countries with Global Disease Detection Regional Centers. J Infect Dis. 2013;208 Suppl 3:S246-54.

29. Agha R, Avner JR. Delayed Seasonal RSV Surge Observed During the COVID-19 Pandemic. Pediatrics. 2021;148(3).

30. Hossain ME, Rahman MZ, Islam MM, Hoque AF, Sumiya MK, Begum MN, et al. Pre COVID-19 molecular epidemiology of respiratory syncytial virus (RSV) among children in Bangladesh. Heliyon. 2022;8(10):e11043.

31. Shawon MSR, Adhikary G, Ali MW, Shamsuzzaman M, Ahmed S, Alam N, et al. General service and child immunization-specific readiness assessment of healthcare facilities in two selected divisions in Bangladesh. BMC Health Serv Res. 2018;18(1):39.

32. Billah MM, Zaman K, Estivariz CF, Snider CJ, Anand A, Hampton LM, et al. Cold-Chain Adaptability During Introduction of Inactivated Polio Vaccine in Bangladesh, 2015. J Infect Dis. 2017;216(suppl_1):S114-s21.

33. Biswas M, Adams SJ, Xing L, Mondal P, Szafron M. Exploring healthcare facilities' readiness for standard precautions in infection prevention and control: a cross-country comparative analysis of six low- and middle-income countries using national cross-sectional surveys. J Glob Health. 2025;15:04205.

34. Pathirana J, Nkambule J, Black S. Determinants of maternal immunization in developing countries. Vaccine. 2015;33(26):2971-7.

35. Lee N, Walsh EE, Sander I, Stolper R, Zakar J, Wyffels V, et al. Delayed Diagnosis of Respiratory Syncytial Virus Infections in Hospitalized Adults: Individual Patient Data, Record Review Analysis and Physician Survey in the United States. J Infect Dis. 2019;220(6):969-79.

36. European Health Management Association (EHMA). THE HEALTH SYSTEM BURDEN OF RESPIRATORY SYNCYTIAL VIRUS (RSV) IN EUROPE. European Health Management Association (EHMA); 2022.

37. Baqui AH, McCollum ED, Saha SK, Roy AK, Chowdhury NH, Harrison M, et al. Pneumococcal Conjugate Vaccine impact assessment in Bangladesh. Gates Open Res. 2018;2:21.

38. Administration USFD. ABRYSVO. In: Administration FD, editor. Washington DC: Government of United States of America; 2023.

39. The Centers for Disease Control and Prevention. Current CDC Vaccine Price List 2025 [Available from: https://www.cdc.gov/vaccines-for-children/php/awardees/current-cdc-vaccine-price-list.html.

40. American Academy of Pediatrics. Nirsevimab (Beyfortus) Product & Ordering Information: Nirsevimab (Beyfortus) Product & Ordering Information; 2024 [

41. Drug.com. Synagis Prices, Coupons, Copay Cards & Patient Assistance 2025 [Available from: https://www.drugs.com/price-guide/synagis.

42. The U.S. Food and Drug Administration. FDA-Approved Drugs: ENFLONSIA™ (clesrovimab-cfor) injection, for intramuscular use Initial U.S. Approval: 2025 2025 [Available from: https://www.accessdata.fda.gov/drugsatfda_docs/label/2025/761432s000lbledt.pdf.

43. World Health Organization. Expanded programme on Immunization (EPI) factsheet 2024: Bangladesh Geneva: World Health Organization,; 2023 [updated 20 August 2024. Available from: https://www.who.int/bangladesh/about-us/publications/i/item/bangladesh-epi-factsheet-2024.

44. Kaufman J, Overmars I, Fong J, Tudravu J, Devi R, Volavola L, et al. Training health workers and community influencers to be Vaccine Champions: a mixed-methods RE-AIM evaluation. BMJ Glob Health. 2024;9(9).

45. Hassan MZ, Haider S, Aleem MA, Islam MA, Shuvo TA, Bhuiya S, et al. Addressing influenza in Bangladesh: closing evidence and policy gaps with strategic interventions. Lancet Reg Health Southeast Asia. 2025;37:100592.

46. Pennisi F, Genovese C, Gianfredi V. Lessons from the COVID-19 Pandemic: Promoting Vaccination and Public Health Resilience, a Narrative Review. Vaccines (Basel). 2024;12(8).

47. Haque R, Moe CL, Raj SJ, Ong L, Charles K, Ross AG, et al. Wastewater surveillance of SARS-CoV-2 in Bangladesh: Opportunities and challenges. Curr Opin Environ Sci Health. 2022;27:100334.

48. World Health Organization. "Crafting the mosaic": a framework for resilient surveillance for respiratory viruses of epidemic and pandemic potential Geneva: World Health Organization; 2023 [Available from: https://www.who.int/publications/i/item/9789240070288.

49. Pebody R, Moyes J, Hirve S, Campbell H, Jackson S, Moen A, et al. Approaches to use the WHO respiratory syncytial virus surveillance platform to estimate disease burden. Influenza Other Respir Viruses. 2020;14(6):615-21.

50. Islam MA, Hassan MZ, Akhtar Z, Bhuiya S, Shuvo TA, Ghosh PK, et al. Leveraging the influenza sentinel surveillance platform for SARS-CoV-2 monitoring in Bangladesh (2020-2024): a prospective sentinel surveillance study. Lancet Reg Health Southeast Asia. 2025;41:100657.

51. Byington CL, Ampofo K, Stockmann C, Adler FR, Herbener A, Miller T, et al. Community Surveillance of Respiratory Viruses Among Families in the Utah Better Identification of Germs-Longitudinal Viral Epidemiology (BIG-LoVE) Study. Clin Infect Dis. 2015;61(8):1217-24.

52. Rios-Guzman E, Simons LM, Dean TJ, Agnes F, Pawlowski A, Alisoltanidehkordi A, et al. Deviations in RSV epidemiological patterns and population structures in the United States following the COVID-19 pandemic. Nat Commun. 2024;15(1):3374.

53. Rogawski McQuade ET, Blake IM, Brennhofer SA, Islam MO, Sony SSS, Rahman T, et al. Real-time sewage surveillance for SARS-CoV-2 in Dhaka, Bangladesh versus clinical COVID-19 surveillance: a longitudinal environmental surveillance study (December, 2019-December, 2021). Lancet Microbe. 2023;4(6):e442-e51.

54. Pang J, Wong JCC, Wulandari SM, Tay M, Karlsson EA, Oktaria V, et al. Wastewater surveillance for early pathogen detection in Asia. Int J Environ Health Res. 2025:1-10.

55. Nourbakhsh S, Shoukat A, Zhang K, Poliquin G, Halperin D, Sheffield H, et al. Effectiveness and cost-effectiveness of RSV infant and maternal immunization programs: A case study of Nunavik, Canada. EClinicalMedicine. 2021;41:101141.

56. Lugnér AK, Mylius SD, Wallinga J. Dynamic versus static models in cost-effectiveness analyses of anti-viral drug therapy to mitigate an influenza pandemic. Health Econ. 2010;19(5):518-31.

57. NHS England. Vaccine Storage and Handling - Cold Chain Policy: NHS England,; 2022 [Available from: https://www.england.nhs.uk/east-of-england/wp-content/uploads/sites/47/2022/07/East-Cold-Chain-Policy-April-2021-v5-6.pdf?utm_source=chatgpt.com.

58. Kumar G, Gupta S. Assessment of cold chain equipments and their management in government health facilities in a District of Delhi: A cross-sectional descriptive study. Indian J Public Health. 2020;64(1):22-6.

59. Lakshmanan K, Liu BM. Impact of Point-of-Care Testing on Diagnosis, Treatment, and Surveillance of Vaccine-Preventable Viral Infections. Diagnostics (Basel). 2025;15(2).

60. Yang Q, Xiao X, Gu X, Liang D, Cao T, Mou J, et al. Surveillance of common respiratory infections during the COVID-19 pandemic demonstrates the preventive efficacy of non-pharmaceutical interventions. Int J Infect Dis. 2021;105:442-7.

61. World Health Organization. WHO GLOBAL MARKET STUDY SEASONAL INFLUENZA VACCINE: World Health Organization; 2024 [Available from: https://cdn.who.int/media/docs/default-source/immunization/mi4a/who_mi4a_global_market_study_seasonal_influenza_vaccine.pdf?sfvrsn=9f24acbf_3&download=true.

62. GAVI: The Vaccine alliance. Gavi Application Process Guidelines. GAVI: The Vaccine alliance; 2023.

63. Levin A, Munthali S, Vodungbo V, Rukhadze N, Maitra K, Ashagari T, et al. Scope and magnitude of private sector financing and provision of immunization in Benin, Malawi and Georgia. Vaccine. 2019;37(27):3568-75.

64. Leal CMSEIM. Beyond COVAX: The Importance of Public-Private Partnerships for Covid-19 Vaccine Delivery to Developing Countries Washington, DC: Center for Strategic and International Studies; 2021 [Available from: https://www.csis.org/analysis/beyond-covax-importance-public-private-partnerships-covid-19-vaccine-delivery-developing.

65. Baral R, Otiang E, Odiyo J, Nyawanda BO, Nyiro JU, Munywoki P, et al. Cost of delivering childhood RSV prevention interventions to the health system in Kenya: a prospective analysis. BMJ Open. 2024;14(11):e084207.

66. Chigiya PT. Respiratory syncytial virus prophylaxis for children in Africa: Challenges, opportunities and public health strategies. Journal of Public Health in Africa. 2025;16(1):1251.

67. World Health Organization. Guidance on an adapted Evidence to Recommendation Process for National Immunization Technical Advisory Groups: World Health Organization; 2022 [Available from: https://iris.who.int/server/api/core/bitstreams/a93402a2-fb21-4dd4-be8d-fe1622480d49/content.

68. The Centers for Disease Control and Prevention. Respiratory Syncytial Virus (RSV) Vaccine Safety USA: US-CDC; 2025 [Available from: https://www.cdc.gov/vaccine-safety/vaccines/rsv.html.

69. Hossain SS HM, Uddin MSG, Reza A, Rahman M, Haq YR. Coverage Evaluation Survey 2019: EPI Bangladesh. In: (CSMR) CfSaMR, editor. DHAKA: GHovernment of Bangladesh; 2020.

70. Islam MS, Kanon N, Huq S, Hassan MS, Islam S, Sarkar H, et al. Incidence and epidemiology of Respiratory Syncytial Virus infections in children in rural Bangladesh: a prospective observational study. medRxiv. 2025:2025.07. 22.25331975.

71. Centre for Diseases Control and Prevention. Healthcare Providers: RSV Immunization for Infants and Young Children: US-CDC; 2023 [Available from: https://www.cdc.gov/vaccines/vpd/rsv/hcp/child.html#:~:text=Resources-,About%20Nirsevimab,severe%20symptoms%20from%20RSV%20infection.

72. Expanded Programme on Immunization (EPI) B. AEFI Surveillance and Response Operational Guideline. In: Welfare MoHaF, editor. 4th ed: Expanded Programme on Immunization (EPI), Directorate General of Health Services, Ministry of Health and Family Welfare, EPI Bhaban, Mohakhali, Dhaka-1212; 2021.

73. Jarrett C, Wilson R, O'Leary M, Eckersberger E, Larson HJ. Strategies for addressing vaccine hesitancy - A systematic review. Vaccine. 2015;33(34):4180-90.

74. Gibson E, Zameer M, Alban R, Kouwanou LM. Community Health Workers as Vaccinators: A Rapid Review of the Global Landscape, 2000-2021. Glob Health Sci Pract. 2023;11(1).

75. Singh P, Dhalaria P, Kashyap S, Soni GK, Nandi P, Ghosh S, et al. Strategies to overcome vaccine hesitancy: a systematic review. Syst Rev. 2022;11(1):78.

Table S2. Distribution Summary of reviewed materials

Category of the reviewed materials	Count (multiple response accepted)	Proportion
Peer-reviewed articles	55	73%
Policy/guidance/technical documents	17	23%
Price / regulatory / market access documents	6	8%
High-income country sources	41	55%
LMIC sources (Bangladesh + LMICs)	34	45%

Table S3. Non-Pharmaceutical Intervention (NPIs) for Control RSV transmission in Bangladesh

Intervention	Description	Evidence of Effectiveness	Considerations for Bangladesh and LMICs
Maintaining Hand Hygiene Practice in household of neonatal and neonatal units in the hospital	Handwashing with soap and water or use of alcohol-based sanitizers to reduce RSV transmission via contaminated surfaces or hands (1-4)	Handwashing reduces respiratory infection rates by 16–21% in community settings (2). Effective in reducing RSV transmission in healthcare settings when combined with other measures (5).	Limited access to clean water and soap in rural areas requires community-level interventions like water sanitation programmes. Public awareness on hand hygiene is critical in densely populated urban areas like Dhaka (6).
Mask-Wearing during visit the household of newborn and mask-wearing by the neonatal parents	Use of face masks (cloth or surgical) by caregivers, healthcare workers, or symptomatic individuals to limit respiratory droplet spread, and reduce transmission of viral infection (1, 3).	Masks reduced RSV transmission during the COVID-19 pandemic in LMICs (7). Effectiveness depends on consistent and proper use of it (6).	Low mask adoption in rural Bangladesh due to cost and cultural practices. Subsidized distribution to the HCP and awareness campaigns needed to promote use at least in the crowded settings and during the RSV season.
Social Distancing with the newborn and their mother	Maintaining physical distance (e.g., 1–2 meters) to reduce close-contact transmission, generally during RSV	Transmission of viruses was lower with physical distancing of 1 m or more, compared with a distance of less	Challenging in densely populated areas like Bangladesh (1,328 people/km²). Targeted distancing in healthcare

	infection season especially in the households or healthcare facilities (1, 4, 6).	than 1 m (aOR 0.18) (8).	settings or during RSV peaks season should be considered.
Improved Ventilation and reduce indoor smoke	Enhancing airflow in indoor settings (e.g., opening windows, using fans) to reduce aerosolized RSV particles (9).	Ventilation improvements in healthcare facilities reduce nosocomial RSV infections (9). There is scarcity of data in community settings.	Poor infrastructure in rural health facilities limits implementation. Low-cost solutions like natural ventilation are viable but require education on benefits.
Isolation of Symptomatic Individuals from the neonates	Isolating children with RSV symptoms to prevent spread in households or communities (6, 10)	Effective in reducing household transmission when symptomatic children are isolated from siblings. Compliance is higher in healthcare settings than communities.	Cultural practices in Bangladesh, such as multigenerational households, do not allow for proper isolation, nor is there sufficient space for it. Community health workers can promote temporary separation strategies.
The family members of newborn should avoid crowd and avoid to attend the newborn in the crowded Avoidance	Reducing attendance at large gatherings or crowded places during RSV season (6).	In studies of high-risk children, residential crowding was associated with a significantly increased likelihood of laboratory-confirmed RSV hospitalisation, with odds ratios ranging from 1.45 to 2.85 (11). Avoiding crowds during these periods reduces transmission risk (3).	Urban slums and public transport in Bangladesh increase crowding. Public campaigns to avoid gatherings during RSV seasons are needed.
Health Education and Awareness of neonatal parents about IPC and viral transmission	Educating communities on RSV symptoms, transmission, and prevention to encourage early care-seeking and NPI adoption (12).	Awareness campaigns improve hand hygiene and care-seeking in LMICs (13). In Bangladesh, education reduces delayed treatment for severe RSV cases.	Low health literacy in rural areas necessitates radio, mobile phone, or community worker-led campaigns (12). Engaging local leaders is key.
School-Based Interventions	Promoting NPIs in schools (e.g., school closure during an influenza pandemic, handwashing stations, mask use,) to reduce RSV spread among children (14).	School closure during an influenza pandemic decreases 28% visits to physician due to respiratory infections (14)	High enrollment in Bangladeshi school offers opportunities for school-based programmes, but resource constraints limit scalability. Instead of closing school, other NPI could be strictly maintained in LMICs like Bangladesh at least during the RSV session (6).

RSV: Respiratory syncytial virus; NPI: Non-pharmaceutical interventions, LMIC: Low and Middle-Income Countries

References of Table S3:

1. World Health Organization. WHO guidelines on hand hygiene in health care Geneva: World Health Organization; 2009 [Available from: https://www.who.int/publications/i/item/9789241597906.
2. The Centers for Disease Control and Prevention. Handwashing Facts: US-CDC; 2024 [Available from: https://www.cdc.gov/clean-hands/data-research/facts-stats/index.html.
3. Dallagiacoma G, Arthur Rhedin S, Odone A, Alfvén T. A comparative analysis of non-pharmaceutical interventions for preventing the respiratory syncytial virus in 30 European countries. Acta Paediatr. 2024;113(6):1388-95.
4. Zhang XL, Zhang X, Hua W, Xie ZD, Liu HM, Zhang HL, et al. Expert consensus on the diagnosis, treatment, and prevention of respiratory syncytial virus infections in children. World J Pediatr. 2024;20(1):11-25.
5. Ross I, Bick S, Ayieko P, Dreibelbis R, Wolf J, Freeman MC, et al. Effectiveness of handwashing with soap for preventing acute respiratory infections in low-income and middle-income countries: a systematic review and meta-analysis. Lancet. 2023;401(10389):1681-90.
6. Hassan MZ, Islam MA, Haider S, Shirin T, Chowdhury F. Respiratory Syncytial Virus-Associated Deaths among Children under Five before and during the COVID-19 Pandemic in Bangladesh. Viruses. 2024;16(1).
7. Sung AD, Sung JAM, Thomas S, Hyslop T, Gasparetto C, Long G, et al. Universal Mask Usage for Reduction of Respiratory Viral Infections After Stem Cell Transplant: A Prospective Trial. Clin Infect Dis. 2016;63(8):999-1006.
8. Chu DK, Akl EA, Duda S, Solo K, Yaacoub S, Schünemann HJ. Physical distancing, face masks, and eye protection to prevent person-to-person transmission of SARS-CoV-2 and COVID-19: a systematic review and meta-analysis. Lancet. 2020;395(10242):1973-87.
9. World Health Organization. Coronavirus disease (COVID-19): Ventilation and air conditioning Geneva: World Health Organization; 2021 [Available from: https://www.who.int/news-room/questions-and-answers/item/coronavirus-disease-covid-19-ventilation-and-air-conditioning.
10. Turner TL, Kopp BT, Paul G, Landgrave LC, Hayes D, Jr., Thompson R. Respiratory syncytial virus: current and emerging treatment options. Clinicoecon Outcomes Res. 2014;6:217-25.
11. Colosia AD, Masaquel A, Hall CB, Barrett AM, Mahadevia PJ, Yogev R. Residential crowding and severe respiratory syncytial virus disease among infants and young children: a systematic literature review. BMC Infect Dis. 2012;12:95.
12. Aiello AE, Coulborn RM, Perez V, Larson EL. Effect of hand hygiene on infectious disease risk in the community setting: a meta-analysis. Am J Public Health. 2008;98(8):1372-81.
13. Loftus MJ, Guitart C, Tartari E, Stewardson AJ, Amer F, Bellissimo-Rodrigues F, et al. Hand hygiene in low- and middle-income countries. Int J Infect Dis. 2019;86:25-30.
14. Heymann A, Chodick G, Reichman B, Kokia E, Laufer J. Influence of school closure on the incidence of viral respiratory diseases among children and on health care utilization. Pediatr Infect Dis J. 2004;23(7):675-7.

Table S4. Adaptation of WHO's Evidence-to-Recommendation (EtR) Framework for Introduction of Maternal RSV Vaccine and Infant Monoclonal Antibody in Bangladesh

Evidence-to-Recommendation Criterion	Evidence	Preliminary decision based on criterion of Bangladesh
**Public Health Problem**	Studies indicate ~20% RSV positivity and ~2% in-hospital mortality among under-five inpatients. Seasonal peaks during the Oct-Dec periods increase paediatric inpatient demand [1-4].	RSV poses a high-priority public health concern warranting targeted preventive interventions.
**Benefits of the Intervention**	Maternal RSV vaccination (RSVpreF) and infant mAb (nirsevimab) show >80% efficacy in preventing severe RSV-associated disease and hospitalisation in clinical trials [5-8]. These interventions protect under-5 during the early months of life when vulnerability is greatest.	Suggested to substantially reduce RSV-related hospitalisations, severity, and mortality in under-5. scarce of country specific data.
**Harms and Safety**	Clinical trials and WHO prequalification assessments report acceptable safety profiles for both RSVpreF vaccine and nirsevimab, with no major safety concerns [9]. Will monitor by PVAE of DGHS.	Benefits outweigh risks; interventions are considered safe for implementation under existing ANC and EPI frameworks.
**Values and Acceptability to Stakeholders**	Experience with maternal tetanus, and COVID-19 vaccines indicates strong community trust in maternal immunization. Stakeholders (MoHFW, DGHS, EPI, WHO, UN agencies) support the introduction of immunization approach [10].	Scarce of country specific data on RSV. Lessons from tetanus, PCV showed high acceptability anticipated among policymakers, healthcare providers, and mothers with appropriate communication strategies.
**Resource Use / Cost-effectiveness**	Annual RSV-associated direct healthcare costs are estimated at ~US$10 million [11]. Global modelling indicates maternal RSV vaccination and infant mAb are cost-effective in LMICs when considering DALYs averted and reduced hospitalisations [11]. Potential support from Gavi could offset introduction costs.	Likely cost-effective; introduction feasible with external financing and integration into existing ANC/EPI delivery platforms. However, there is lack of Bangladesh specific data on cost-effectiveness modelling.
**Equity and Human Rights**	RSV disproportionately affects under-5 in low-income and rural areas with limited access to tertiary care. Maternal immunisation can extend protection to these populations via ANC outreach [11].	Intervention likely to enhance health equity and uphold the right to preventive healthcare for vulnerable populations such as preterm under-5 who had higher risk of RSV infections [12].
**Feasibility**	Bangladesh's EPI and ANC systems have successfully delivered maternal tetanus, PCV-10, and COVID-19 vaccines [13]. Cold-chain infrastructure and trained personnel are available; mAb may require additional logistics planning.	Operationally feasible; integration into ANC services and EPI systems achievable with minor logistical and training facility enhancements.

Evidence-to-Recommendation Criterion	Evidence	Preliminary decision based on criterion of Bangladesh
**Balance of Consequences**	The magnitude of benefits (disease reduction, mortality prevention, cost savings) outweighs potential risks and resource constraints. The intervention aligns with national health goals and WHO recommendations [10].	The overall balance of consequences strongly favors introduction of maternal RSV vaccination, followed by infant mAb rollout.
**Overall Recommendation**	Based on available evidence and contextual factors, the NITAG and MoHFW may recommend phased introduction of maternal RSV vaccine integrated into ANC, supported by strengthened surveillance and evaluation mechanisms and mAb into EPI.	Recommend introduction of maternal RSV vaccination as a priority intervention; consider future mAb inclusion pending resources and programmatic readiness.

References:

1. Homaira N, Luby SP, Hossain K, Islam K, Ahmed M, Rahman M, et al. Respiratory Viruses Associated Hospitalization among Children Aged <5 Years in Bangladesh: 2010-2014. PLoS One. 2016;11(2):e0147982.
2. Reller ME, Mehta K, McCollum ED, Ahmed S, Anderson J, Roy AD, et al. Viral Acute Lower Respiratory Tract Infections (ALRI) in Rural Bangladeshi Children Prior to the COVID-19 Pandemic. Influenza Other Respir Viruses. 2024;18(12):e70062.
3. Saha S, Saha S, Kanon N, Hooda Y, Islam MS, Islam S, et al. Health-care burden related to respiratory syncytial virus in a resource-constrained setting: a prospective observational study. Lancet Glob Health. 2025;13(6):e1072-e81.
4. Begum MN, Karim Y, Jubair M, Khair S, Tony S, Patwary M, et al. High Burden of Respiratory Syncytial Virus Among Bangladeshi Hospitalized Children Under Five2024.
5. Government of United Kingdom. RSV vaccination of pregnant women for infant protection: information for healthcare practitioners. In: Agency UKHS, editor. 2025.
6. Administration USFD. ABRYSVO. In: Administration FD, editor. Washington DC: Government of United States of America; 2023.
7. The U.S. Food and Drug Administration. FDA-Approved Drugs: ENFLONSIA™ (clesrovimab-cfor) injection, for intramuscular use Initial U.S. Approval: 2025 2025 [Available from: https://www.accessdata.fda.gov/drugsatfda_docs/label/2025/761432s000lbledt.pdf.
8. Drysdale SB, Cathie K, Flamein F, Knuf M, Collins AM, Hill HC, et al. Nirsevimab for Prevention of Hospitalizations Due to RSV in Infants. N Engl J Med. 2023;389(26):2425-35.
9. The Centers for Disease Control and Prevention. Respiratory Syncytial Virus (RSV) Vaccine Safety USA: US-CDC; 2025 [Available from: https://www.cdc.gov/vaccine-safety/vaccines/rsv.html.
10. Barsosio HC, Bont LJ, Groome MJ, Karron RA, Kragten-Tabatabaie L, Madhi SA, et al. How Gavi support for RSV immunisation will advance health equity. Lancet. 2025.
11. Nourbakhsh S, Shoukat A, Zhang K, Poliquin G, Halperin D, Sheffield H, et al. Effectiveness and cost-effectiveness of RSV infant and maternal immunization programs: A case study of Nunavik, Canada. EClinicalMedicine. 2021;41:101141.

12. Islam MS, Kanon N, Huq S, Hassan MS, Islam S, Sarkar H, et al. Incidence and epidemiology of Respiratory Syncytial Virus infections in children in rural Bangladesh: a prospective observational study. medRxiv. 2025:2025.07. 22.25331975.
13. Hossain SS HM, Uddin MSG, Reza A, Rahman M, Haq YR. Coverage Evaluation Survey 2019: EPI Bangladesh. In: (CSMR) CfSaMR, editor. DHAKA: GHovernment of Bangladesh; 2020.

Figure S1. Step-by-step introduction of the RSV maternal vaccine and monoclonal antibody for children under-5 for strengthening RSV control program in Bangladesh.

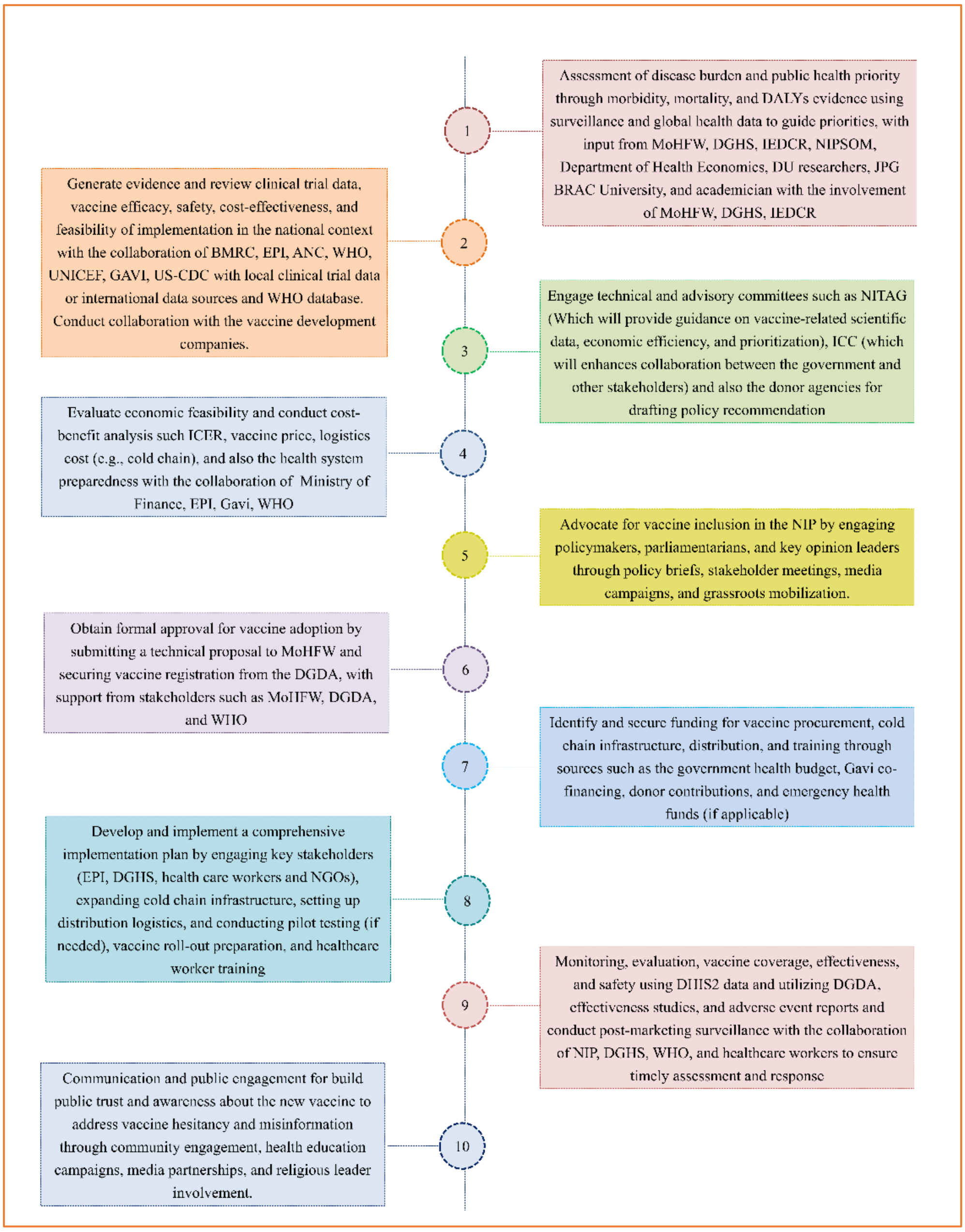

BMRC – Bangladesh Medical Research Council, DALYs – disability-adjusted life years, DGDA – Directorate General of Drug Administration, DGHS – Directorate General of Health Services, EPI – Expanded Programme on Immunization, ICC – Interagency Coordination Committee, ICER – incremental cost-effectiveness ratio, IEDCR – Institute of Epidemiology Disease Control and Research, JPG – BRAC James P Grant School of Public Health, BRAC University, MoHFW – Ministry of Health and Family Welfare, NIP – National Immunization Programme, NIPSOM – National Institute of Preventive and Social Medicine, NITAG – National Immunization Technical Advisory Group, UN – United Nations, WHO – World Health Organization.
